# Sp1-mediated microRNA-182 expression regulates lung cancer progression

**DOI:** 10.18632/oncotarget.1608

**Published:** 2014-01-25

**Authors:** Wen-Bin Yang, Ping-Hsin Chen, Tsung-I Hsu, Tzu-Fun Fu, Wu-Chou Su, Hungjiun Liaw, Wen-Chang Chang, Jan-Jong Hung

**Affiliations:** ^1^ Institute of Bioinformatics and Biosignal Transduction, College of Bioscience in Biotechnology, National Cheng Kung University, Tainan 701, Taiwan; ^2^ Department of Pharmacology, College of Medicine, National Cheng Kung University, Tainan 701, Taiwan; ^3^ Center for Infectious Disease and Signal Transduction Research, National Cheng Kung University, Tainan 701, Taiwan; ^4^ Department of Medical Laboratory Science and Biotechnology, College of Medicine, National Cheng Kung University, Tainan 701, Taiwan; ^5^ Department of Internal Medicine, College of Medicine and Hospital, National Cheng Kung University, Tainan 701, Taiwan; ^6^ Department of Life Sciences, College of Bioscience in Biotechnology, National Cheng Kung University, Tainan 701, Taiwan; ^7^ Graduate Institute of Medical Sciences, College of Medicine, and Center for Neurotrauma and Neuroregeneration, Taipei Medical University, Taipei 110, Taiwan

**Keywords:** Sp1, miR-182, FOXO3, Lung cancer

## Abstract

Our recent study indicated that overexpression of Sp1 enhances the proliferation of lung cancer cells, while represses metastasis. In this study, we found that the transcriptional activity of FOXO3 was increased, but its protein levels decreased following Sp1 expression. Sp1 increased expression of miR-182, which was then recruited to the 3'-untranslated region of FOXO3 mRNA to silence its translational activity. Knockdown of miR-182 inhibited lung cancer cells growth, but enhanced the invasive and migratory abilities of these cells through increased N-cadherin expression. Repression of FOXO3 expression in the miR-182 knockdown cells partially reversed this effect, suggesting that miR-182 promotes cancer cell growth and inhibits cancer metastatic activity by regulating the expression of FOXO3. The expression of several cancer metastasis-related genes such as ADAM9, CDH9 and CD44 was increased following miR-182 knockdown. In conclusion, in the early stages of lung cancer progression, Sp1 stimulates miR-182 expression, which in turn decreases FOXO3 expression. This stimulates proliferation and tumor growth. In the late stages, Sp1 and miR-182 decline, thus increasing FOXO3 expression, which leads to lung metastasis.

## INTRODUCTION

Post-transcriptional regulation plays an important role in diverse cellular processes such as development, neurogenesis, and cancer progression [1-3]. MicroRNAs (miRNAs) have emerged as important post-transcriptional regulators that inhibit mRNA translation or induce mRNA cleavage by base pairing with a seed region in the 3'-untranslated region (3'-UTR) of target genes [4, 5]. Recent studies have shown that dysregulation of miRNAs contributes to the initiation, progression, metastasis, and drug resistance of cancer [6, 7]. For example, miR-200c targets Kras to regulate Kras expression during tumorigenesis [8]. Furthermore, several upregulated and downregulated miRNAs have been identified in lung cancer, the most frequently diagnosed cancer and the most common cause of cancer-related death worldwide [9-11]. Identification of early-detection biomarkers and precise diagnosis are necessary if lung cancer patients are to receive efficacious therapeutic treatment quickly. Several factors such as USP17 have been identified as potential biomarkers for lung cancer [12, 13]. Circulating miRNAs could also serve as useful clinical biomarkers for the screening of high-risk populations and the detection solid tumors in the early stages of cancer progression [14, 15]. miRNAs offer new targets for cancer therapy [16, 17]. Therefore, a detailed understanding of the mechanisms underlying miRNA production and function is important.

Identification of miRNA target genes and the use of gene set enrichment analysis have clarified the function role of miRNAs. However, the molecular mechanisms that regulate of miRNA biogenesis are still largely unknown. Recent studies have shown that transcription factors (TFs) regulate not only the expression of protein-encoding genes, but also miRNA biogenesis through RNA polymerase II-dependent transcription [18]. Several TFs including p53, c-myc, and HIF1α that directly recognize miRNA promoters and regulate miRNA transcription have been reported [19-21].

Specificity protein 1 (Sp1), which belongs to the specificity protein/ Krüppel-like family, was the first TF identified in mammalian cells. Sp1 contains three Cys_2_His_2_-type zinc finger DNA binding motifs that recognize GC-rich promoter sequences [22]. Sp1 regulates thousands of coding genes, such as those encoding cyclin A2, p21^cip1/waf1^, E-cadherin and Sp1 itself. These genes are involved in a variety of physiological processes including cell cycle progression and cell migration [23-26]. Sp1 also regulates the expression of noncoding genes. Sp1 forms a complex with NF-κB to downregulate miR-29b expression through the recruitment of histone deacetylase (HDAC) 1 and HDAC3 in leukemia and thereby contributes to the growth of leukemia cells [27]. Sp1 also forms a complex with HDAC4 to downregulate miR-200a expression in hepatocellular carcinoma and contributes to cell proliferation and migration [28]. In addition, Sp1 is an activator of miR-34c, miR-132 and miR-365 expression [29-31]. However, no studies have assessed whether Sp1 regulates the expression of miRNAs involved in lung tumorigenesis. Because the accumulation of Sp1 is required for lung tumor growth, further investigation of Sp1-mediated miRNA regulation is needed.

In this study, we showed that Sp1 suppressed FOXO3 expression via post-transcriptional regulation. To elucidate whether miRNAs were involved in this process, we used a systematic screening approach to identify Sp1-regulated miRNAs. We identified a novel Sp1-regulated miRNA, miR-182, in lung cancer cells and demonstrated that Sp1 downregulated FOXO3 expression by upregulating miR-182 expression. Our results show that miR-182 functions as an oncomiR to enhance cancer cell proliferation, and acts as a tumor suppressor to inhibit cancer metastasis.

## RESULTS

### Sp1 regulates miR-182 expression

Our previous studies demonstrated that Sp1 is involved in Kras^G12D^-induced lung tumorigenesis [23, 32]. Using cDNA microarray analysis, we found that Sp1 increased oncogene expression and decreased tumor suppressor gene expression. In the present study, we initially used software to analyze the promoters of all identified miRNAs. According to the miRBase database, the human genome contains 1600 miRNA genes. We investigated whether Sp1 participates in the regulation of intergenic miRNAs. First, we screened the upstream (-1 kb) flanking sequences of intergenic miRNAs. Using the TFSEARCH program, we identified 205 intergenic miRNAs that contained potential binding sites for Sp1. Because Sp1 is upregulated in lung cancer and the expression of its target genes is altered, we next examined the expression of these miRNAs in lung cancer. According to previous studies, the expression patterns of 22 miRNAs differed significantly in lung cancer tissue and normal lung tissue (Supplementary Table S1). In most of these studies, miR-182, which contains two putative Sp1 binding sites within its upstream region, was upregulated in lung cancer.

**Figure 1:**
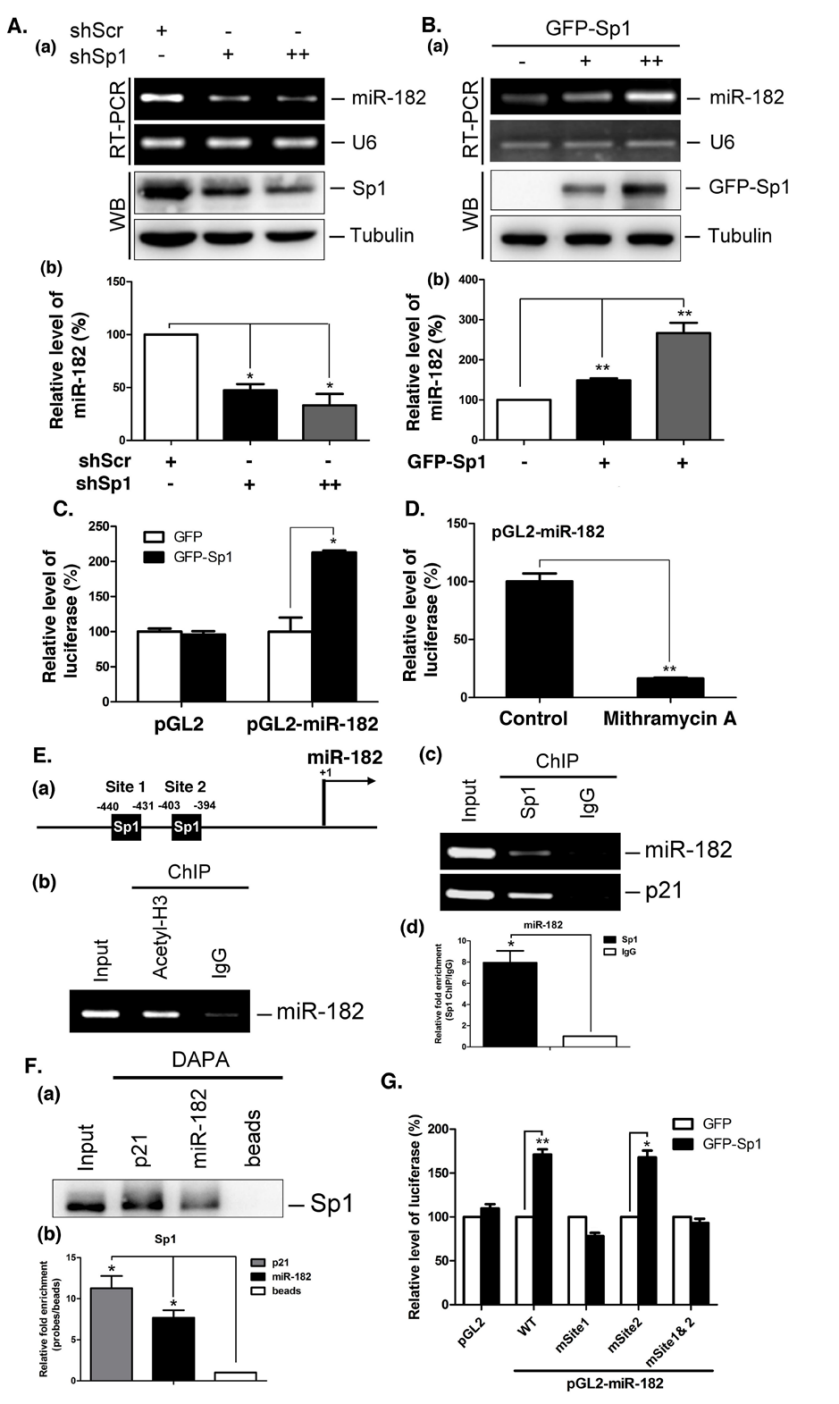
Sp1 regulates miR-182 expression (A) Scramble (shScr) and different doses of Sp1 shRNAs (shSp1) were transfected into A549 for 48 h. The miR-182 level was determined by stem-loop RT-PCR. U6 served as the internal control (panel a). Data were quantified after three independent experiments (panel b). (B) Different titer of adeno-GFP-Sp1 virus was infected IMR-90 cells for 48 h. The miR-182 level was determined by stem-loop RT-PCR (panel a). Data were quantified after three independent experiments (panel b). (C) Plasmids, pGL2 or pGL2-miR-182 (-1000/+50), and GFP or GFP-Sp1 were co-transfected into H1299 cells for 24h. Cells were harvested to study the luciferase activity. Data were quantified after three independent experiments. (D) The plasmids, pGL2 or pGL2-miR-182, were transfected into H1299 cells with mithramycin A treatment for 24 h. Cells were harvested for luciferase activity assays. (E) Schematic diagram indicates the location of putative Sp1 binding sites on miR-182 promoter region (panel a). ChIP assays were performed with anti-acetyl-H3 (panel b), and anti-Sp1 antibodies (panel c). DNA was extracted for PCR with miR-182 and p21 primers. Data were quantified after three independent experiments (panel d). (F) A549 cells were harvested for DAPA with a biotin-conjugated p21 and miR-182 promoter probes, and samples were analyzed by Western blotting using anti-Sp1 antibodies (panel a). Data were quantified after three independent experiments (panel b). (G) Plasmids, GFP or GFP-Sp1, were co-transfected with pGL2, pGL2-miR-182 WT or mutation plasmids into H1299 cells for 24 h, and then cells were harvested for luciferase activity assays. Data are representative of three independent experiments, each of which was performed in triplicate, and presented as the mean ± SEM. The level of statistical significance determined by *t*-test (*, p<0.05; **, p<0.01).

When we examined miR-182 expression, we found that miR-182 was decreased in Sp1-knockdown cells, but increased in IMR-90 cells that overexpressed GFP-Sp1 (Figure 1A and 1B), suggesting that Sp1 positively regulates miR-182 expression. Luciferase activity driven by the miR-182 promoter increased in H1299 cells overexpressing GFP-Sp1 (Figure 1C), whereas luciferase activity decrease in cells treated with an Sp1 inhibitor, mithramycin A (Figure 1D). These results suggested that Sp1 is involved in miR-182 transcriptional activation. Using the TFSEARCH software, we analyzed the miR-182 promoter and identified two putative Sp1 binding elements. Consequently, recruitment of Sp1 to the miR-182 promoter was examined (Figure 1E and 1F). Acetyl-histone3 was recruited to the Sp1 binding elements, indicating that the region could recruit TFs (Figure 1E, panel b). Sp1 was also recruited to the miR-182 promoter (Figure 1E, panel c and panel d, and 1F). When the Sp1 binding element at site 1 was mutated, luciferase activity driven by the miR-182 promoter was abolished, but no change was observed when the other Sp1 binding site was mutated, indicating that the Sp1 binding element at site 1 is important for the Sp1-mediated expression of miR-182 (Figure 1G).

**Figure 2:**
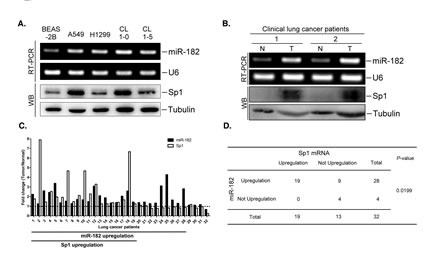
The miR-182 level correlates to Sp1 level Total RNA and cell lysates were prepared from indicated cell lines (A) or from clinical lung tissues of lung cancer patients (B). The miR-182 level was determined by stem-loop RT-PCR and Sp1 level was studied by Western blotting with anti-Sp1 antibodies. U6 and tubulin served as the internal control. (C) Total RNA and cell lysates were prepared from 32 paired normal lung tissues and lung adenocarcinoma samples. The miR-182 level was studied by stem-loop RT-PCR and Sp1 levels were studied by RT-PCR. (D) The relationship between Sp1 and the miR-182 level in the 32 lung cancer samples was statistically analyzed using Fisher's exact test.

Because Sp1 is highly expressed in lung cancer, we studied the expression of Sp1 and miR-182 in various lung cancer cell lines and patient samples (Figure 2). Compared with normal human lung cells (BEAS-2B), lung cancer cell lines expressed higher levels of miR-182 (Figure 2A). We also assessed the correlation between the miR-182 and Sp1 expression patterns. Sp1 levels in clinical lung tissue samples were highly elevated in the tumorous sections of the lung, accompanied by increased expression of miR-182 (Figure 2B). To confirm this result, Sp1 and miR-182 levels were measured in 32 lung cancer patients. Sp1 and miR-182 were upregulated by more than 1.3-fold in 59.4% of the lung adenocarcinoma specimens when compared to expression in normal tissue (Figure 2C). These results indicate that Sp1 expression positively correlates with miR-182 expression (Figure 2D).

### miR-182 increases lung tumor growth

**Figure 3:**
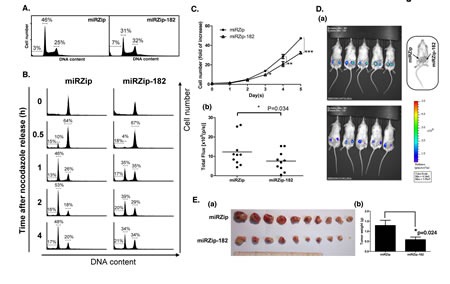
miR-182 increases cancer cell proliferation (A) The miRZip and miRZip-182 stably expressed H1299 cells were fixed with 70% ethanol, and stained with propidium iodide for cell cycle analysis by FACS. (B) Mitotic cells were released into growth by removing nocodazole, then fixed at indicated time points for cell cycle progression assay by FACS. (C) The growth rates of miRZip and miRZip-182 stably expressed H1299 cells were calculated by cell counting within 5 days. Data are representative of six independent experiments, and presented as the mean ± SEM. (D) Bioluminescent imaging was performed on 10 severe combined immunodeficient (SCID) mice implanted with miRZip and miRZip-182 stable expression H1299 cells (10^6^ cells/mouse) at day 14 (panel a), then image signal was analyzed using Living Image software and presented as total flux measurements in photons/second (panel b). (E) Tumors from SCID mice implanted with miRZip and miRZip-182 stable expression H1299 cells for 4 weeks are shown (panel a), and tumor weights were analyzed (panel b). Data are representative of ten independent experiments and are presented as the mean ± SEM. The level of statistical significance determined by *t*-test (*, p<0.05; **, p<0.01; ***, p<0.001).

The data shown in Figure 1 indicated that Sp1 regulated miR-182 expression during lung tumorigenesis. To identify the specific gene targets of miR-182, we searched public miRNA target prediction databases (miRDB, miRWalk, and TargetScanHuman) for candidate target genes. By combining the data from these three databases, we identified 161 genes potentially regulated by miR-182 (Supplementary Figure S1A). Moreover, pathway analysis using Ingenuity software indicated that the cellular growth and proliferation pathway had the highest score when the association of these 161 genes with biological pathways was examined. This suggests that miR-182 may play a functional role in cancer-associated processes (Supplementary Figure S1B). Indeed, when miR-182 was knocked down with miRZip-182 shRNA, the percentage of cells in G2/M and sub-G1 phases increased, suggesting that miR-182 positively regulated cell cycle progression in the lung cancer cells (Figure 3A). To further elucidate miR-182's effect on the cell cycle, cells were synchronized at prometaphase using nocodazole treatment. After removing nocodazole, more miRZip-182 than miRZip cells remained in G2/M phase, providing further evidence that miR-182 positively regulates cell cycle progression (Figure 3B). Consistently, knockdown of miR-182 expression inhibited cell growth (Figure 3C).

To confirm the effect of miR-182 on tumor formation, cells stably expressing with miRZip or miRZip-182 were implanted into SCID mice, and tumor growth was monitored *in vivo* (Figure 3D). The miRZip lentivector contains a copGFP gene, and the GFP signal in miRZip-182-expressing cells was lower than that in miRZip control cells (Figure 3D). Furthermore, tumor volume and tumor weight were also lower in miRZip-182-implanted mice than in miRZip-implanted mice (N = 10 per group) (Figure 3E). These results suggest that miR-182 overexpression facilitates lung tumor growth *in vivo*.

### Sp1 inhibits FOXO3 expression by inducing miR-182 expression

**Figure 4:**
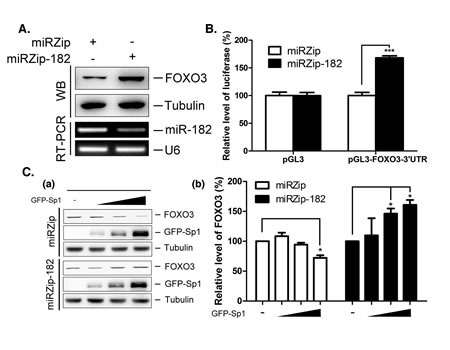
Regulation of FOXO3 by miR-182 and Sp1 (A) Lenti-miRZip and lenti-miRZip-182 viruses were infected into H1299 for 96 h individually. FOXO3 level was studied by Western blotting with anti-FOXO3 antibodies and miR-182 level was studied by stem-loop RT-PCR. (B) Plasmids, pGL3 and pGL3-FOXO3-3'UTR, were transfected into miRZip and miRZip-182 stably expressed H1299 cells for 24 h, and then cells were harvested for luciferase activity assays. (C) Different doses of GFP-Sp1 adenovirus were infected into the miRZip and miRZip-182 stably expressed H1299 cells for 48 h. FOXO3 level was studied by Western blotting using anti-FOXO3 antibodies (panel a). Quantitative results from three independent experiments are shown (panel b). The level of statistical significance was determined by *t*-test (*, p<0.05; ***, p<0.001).

To investigate the molecular mechanism underlying miR-182-mediated cancer cell proliferation, we studied an important miR-182 target gene, FOXO3. FOXO3 expression was higher in cells stably expressing miRZip-182 than in control cells (Figure 4A). Knockdown of miR-182 expression enhanced the luciferase activity of a pGL3 vector containing the 3′-UTR of FOXO3 (Figure 4B), indicating that miR-182 downregulated FOXO3 expression. Further, to determine whether Sp1 downregulated FOXO3 expression through miR-182, GFP-Sp1 was expressed in cells stably expressing miRZip-182 (Figure 4C). Overexpression of GFP-Sp1 reduced FOXO3 protein expression in miRZip stable cells, but increased FOXO3 levels in miRZip-182-expressing cells, implying that different effect of Sp1 is existed on the regulation of FOXO3 expression.

**Figure 5:**
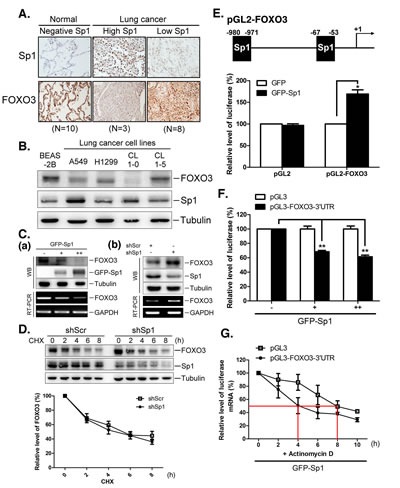
Sp1 negatively regulates FOXO3 expression through regulating miR-182 (A) The Sp1 and FOXO3 levels in clinical lung tissue samples were studied by IHC staining using antibodies against Sp1 and FOXO3, respectively. (B) Cell lysates were harvested from various cell lines for Western blotting using antibodies against FOXO3 and Sp1, and tubulin as an internal control. (C) Adeno-GFP-Sp1 viruses were infected into IMR-90 cells for 48 h, and FOXO3 mRNA and protein were studied by RT-PCR and Western blotting, respectively. GAPDH served as the internal control (panel a). Scramble and Sp1 shRNAs were transfected into H1299 for 48 h, then FOXO3 mRNA and protein levels were studied by RT-PCR and Western blotting (panel b). (D) Scramble and Sp1 shRNAs were transfected into H1299 for 48 h, and then cells were harvested at indicated time points following cycloheximide treatment for studying the Sp1 and FOXO3 levels with Western blotting. The levels of FOXO3 protein from three independent experiments were quantified using tubulin as an internal control. (E) Plasmids, pGL2 or pGL2-FOXO3 (-1000/+50), were cotransfected with GFP or GFP-Sp1 into H1299 cells for 24 h, then cell lysates were harvested for luciferase activity assays. (F) Adeno-GFP-Sp1 viruses were infected into H1299 cells for 24 h, and cells were then transfected with pGL3 or pGL3-FOXO3-3'UTR plasmid for 24 h. Cells lysates were harvested for luciferase activity assays. (G) H1299 cells, which were infected with GFP-Sp1 adenovirus for 24 h, were then transfected with pGL3 or pGL3-FOXO3-3'UTR plasmid for 24 h. Total RNA was extracted at various time points following actinomycin D treatment. The mRNA levels of luciferase were determined by using quantitative RT-PCR and quantified using GAPDH as an internal control. Data are representative of three independent experiments, each of which was performed in triplicate, and presented as the mean ± SEM. The level of statistical significance determined by *t*-test (*, p<0.05; **, p<0.01).

Therefore, we further investigated the relationship between Sp1 and miR-182 in the context of FOXO3 regulation. The expression of Sp1 and FOXO3 in patients with lung cancer was examined (Figure 5A). In normal tissue samples, Sp1 levels were low, and FOXO3 levels were high. In tumor tissue samples, two Sp1 expression patterns, i.e. high and low Sp1 expression, were identified. Samples with higher Sp1 levels exhibited lower FOXO3 levels, whereas samples with lower Sp1 levels exhibited higher FOXO3 levels, suggesting that there is an inverse correlation between Sp1 and FOXO3 levels in lung specimen (Figure 5A). The levels of FOXO3 and Sp1 in the lung cancer cell lines, A549, H1299, CL 1-0, and CL 1-5, were studied (Figure 5B). Higher levels of Sp1 expression were accompanied by lower levels of FOXO3 expression in A549 and CL 1-0 cells, and lower levels of Sp1 expression were accompanied by higher levels of FOXO3 expression in H1299 and CL 1-5 cells, suggesting that there is an inverse correlation between Sp1 and FOXO3 expression in lung tumorigenesis. Overexpression of GFP-Sp1 decreased FOXO3 mRNA and protein levels in a dose-dependent manner (Figure 5C, panel a), whereas knockdown of Sp1 expression increased FOXO3 mRNA and protein levels (Figure 5C, panel b). These results indicate that Sp1 negatively regulates FOXO3 expression.

Next, we investigated the mechanism by which Sp1 regulates FOXO3 expression. FOXO3 protein half-life was studied after Sp1 knockdown. Knockdown of Sp1 expression did not affect FOXO3 protein stability (Figure 5D). We then constructed a luciferase reporter construct containing the FOXO3 promoter (-1000/+50) to study the effect of Sp1 on the promoter-mediated transcription of FOXO3 (Figure 5E). GFP-Sp1 overexpression significantly enhanced the luciferase activity, indicating that Sp1 positively regulated FOXO3 transcription (Figure 5E). However, FOXO3 mRNA and protein levels decreased, as shown in Figure 5C. The data shown in Figure 1 indicated that Sp1 increased miR-182 expression, which suggests that post-transcriptional processing contributes to the regulation of FOXO3 expression. Thus, the 3'-UTR of FOXO3 might play an important role in stabilizing FOXO3 mRNA and in FOXO3 translation. Consequently, a luciferase reporter construct containing the 3′-UTR of FOXO3 was generated. GFP-Sp1 overexpression reduced the luciferase activity (Figure 5F). Furthermore, the stability of the luciferase mRNA containing the 3′-UTR sequence of FOXO3 decreased dramatically upon GFP-Sp1 overexpression (Figure 5G). These results indicate that Sp1 regulates FOXO3 expression through transcriptional and post-transcriptional regulation, with a net negative effect on FOXO3 expression.

### miR-182 inhibits lung cancer metastasis activity

**Figure 6:**
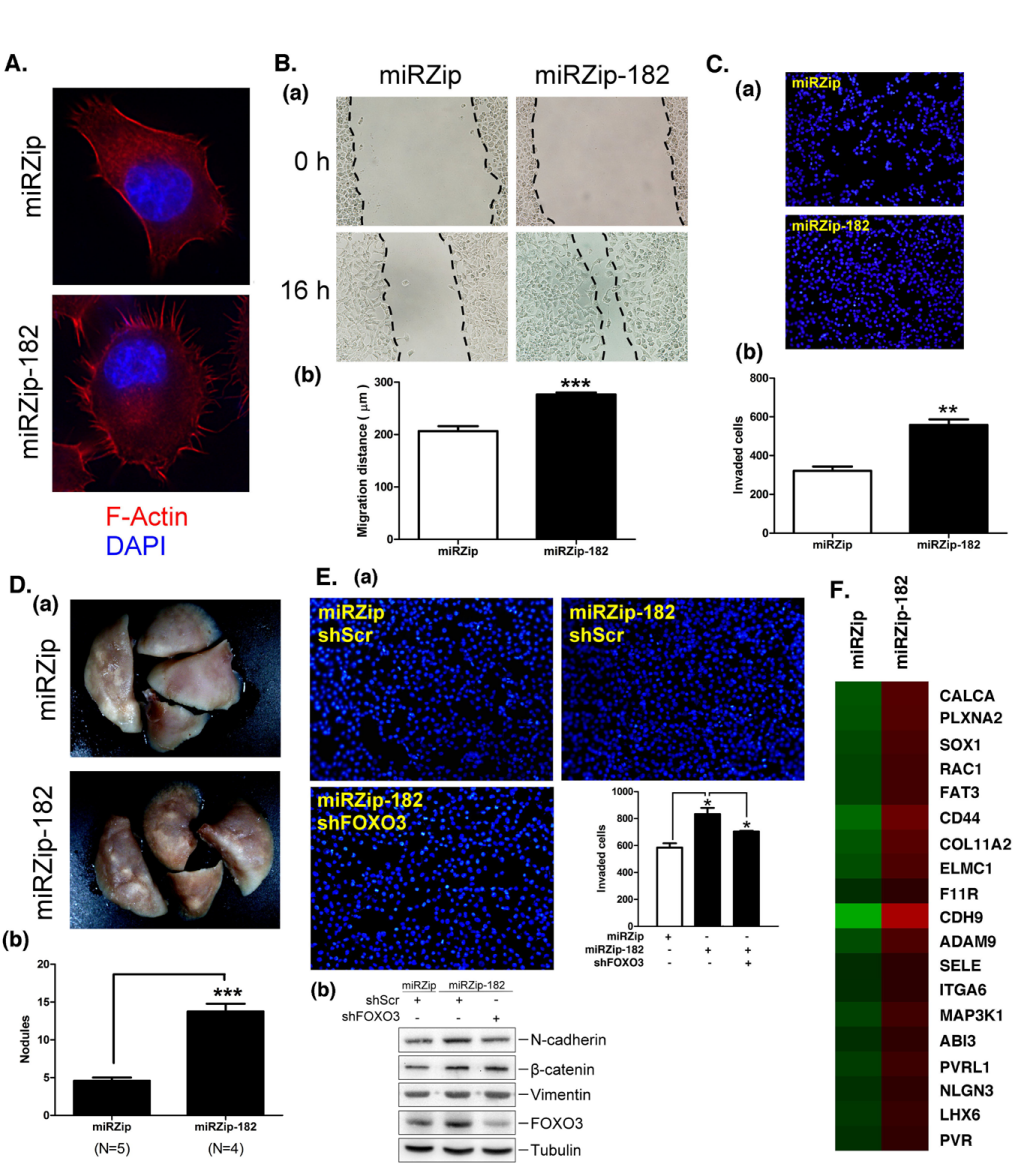
miR-182 attenuates lung cancer cell metastasis (A) Immunofluorescent staining of Alexa Fluor 568-conjugated phalloidin that is a high-affinity probe for F-actin (red) in miRZip and miRZip-182 stably expressed H1299 cells. DNA was stained with DAPI (blue). Stained cells were photographed under a fluorescence microscope at x 600 magnification. (B) Confluent monolayers of miRZip or miRZip-182 stably expressed H1299 cells were wounded and incubated for an additional 16 h (panel a). Migratory area was calculated for quantification (panel b). (C) The migration activities of H1299 cells (2 x 10^4^) expressing miRZip or miRZip-182 were studied by Transwell chambers. (D) The miRZip or miRZip-182 stably expressed H1299 cells (4 x 10^6^) were suspended in 100 μl of PBS and injected into the lateral tail vein of SCID mice. After 8 weeks, all mice were killed and the number of pulmonary tumor nodules was calculated after fixation of lungs with 4% formaldehyde for 48 h (panel a), and the number of pulmonary metastatic tumor nodules was counted (panel b). (E) FOXO3 and miR-182 in H1299 cells were knockdown by shFOXO3 and miRZip-182 respectively, and then migration of cells (3 x 10^4^) was studies by Transwell chambers (panel a). In addition, cell lysates were harvested from FOXO3 and miR-182 knockdown cells for Western blotting using antibodies against N-cadherin, β-catenin, vimentin, FOXO3 and tubulin (panel b), respectively. (F) Heat map of the 19 of genes from miRZip and miRZip-182 microarray data, the red color represents genes that are upregulated and the green color represents genes that are downregulated. The level of statistical significance determined by *t*-test (*, p<0.05; **, p<0.01; ***, p<0.001).

The data shown in Figure 3 indicated that miR-182 positively regulated lung cancer cell growth. Therefore, the role of miR-182 in lung cancer metastasis was studied (Figure 6). The morphology of miRZip-182 cells was markedly altered: circular structures of actin filaments were absence and pseudopodia were enriched, suggesting that miR-182 decreased the cells' migratory ability (Figure 6A). Indeed, knockdown of miR-182 expression increased the migration ability of lung cancer cells, suggesting that miR-182 inhibits lung cancer migration (Figure 6B). Moreover, transwell migration assays showed that knockdown of miR-182 expression enhanced cell's invasive capacity (Figure 6C). In mice injected with miRZip-182-treated cells, the knockdown of miR-182 expression also increased the number of nodules in the lung, suggesting that miR-182 represses metastatic ability *in vivo* (Figure 6D). The effects of miR-182 knockdown were partially reversed by knockdown of FOXO3, suggesting that miR-182 functions as a suppressor of lung cancer metastasis by repressing FOXO3 expression (Figure 6E, panel a). The endothelial-mesenchymal transition (EMT) marker, N-cadherin, increased after miR-182 knockdown, but this effect was abolished by FOXO3 knockdown. Thus, miR-182 might repress lung cancer metastasis by decreasing the expression of N-cadherin (Figure 6E, panel b). However, the expression of other genes regulated by miR-182 might also play a role in metastasis (Figure 6F and Supplementary Figure S3). Therefore, we generated gene expression profiles using microarray analysis. Functional grouping analysis using DAVID bioinformatics resources showed that 19 of the genes differentially regulated by miR-182 knockdown were related to cell migration. The expression of these genes was increased in miR-182-knockdown cells, indicating that they are potential targets of miR-182 (Figure 6F). Many metastasis-related genes such as CD44, CDH9 and ADAM9 were upregulated after the knockdown of miR-182 expression (Figure 6F).

## DISCUSSION

**Figure 7:**
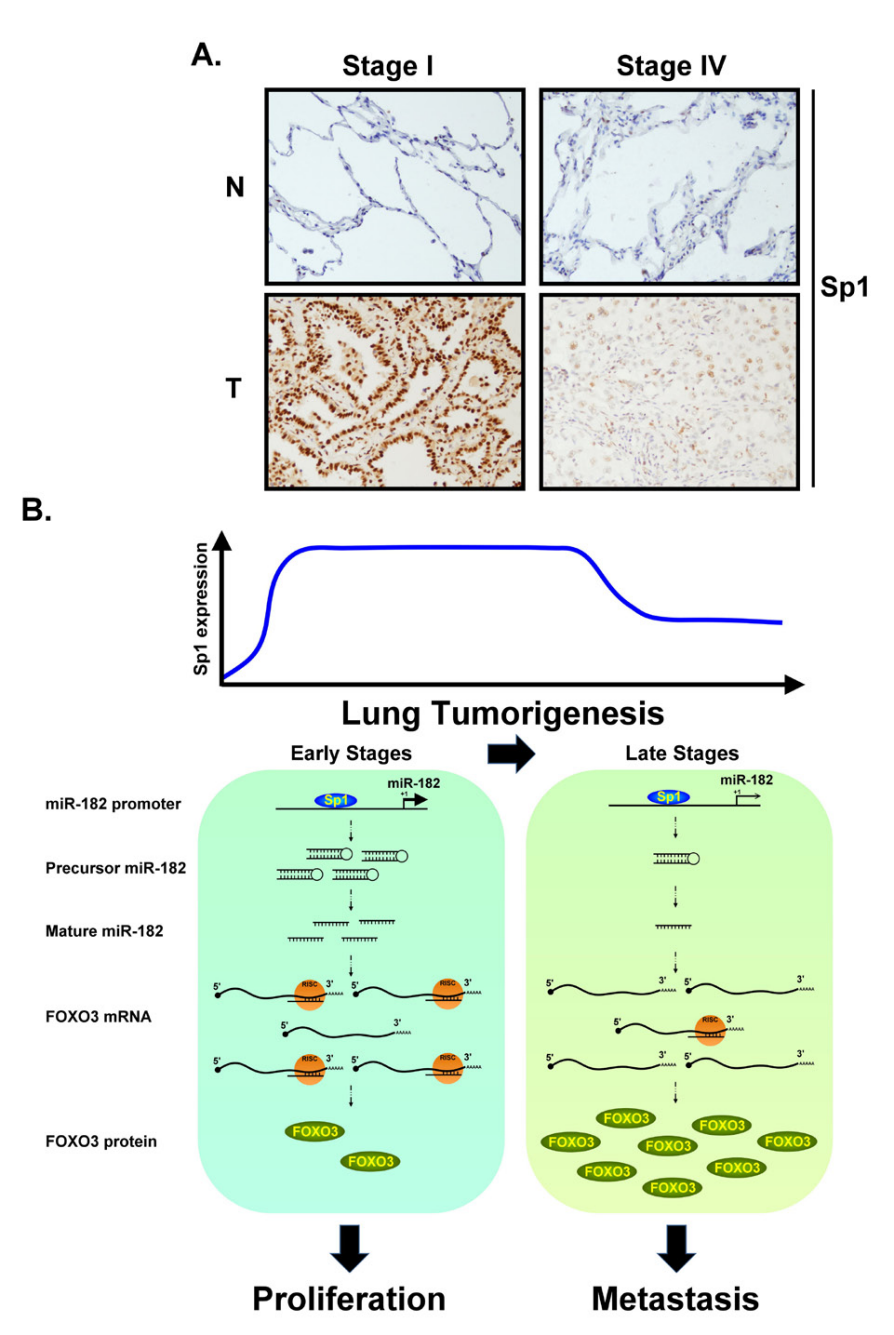
(A) Clinical samples from lung cancer patients of stage I and IV were used to study the Sp1 level by IHC staining with anti-Sp1 antibodies (B) Schematic diagram illustrates Sp1 regulates miR-182 to silence FOXO3 expression in early and late stages of lung cancer progression.

Our recent studies showed that Sp1 increased the growth of lung cancer cells, but inhibits metastatic activity [23, 32]. In the present study, we found that Sp1, which accumulated in the early stages of cancer, positively regulated miR-182 gene expression to silence FOXO3 expression and thereby promote cancer cell growth. In addition, decreased levels of Sp1 in the late stages of cancer increased the expression of FOXO3 and N-cadherin, leading to cancer metastasis (Figure 7).

Sp1 functions as a transcriptional activator by recruiting p300 to its target genes and as a repressor by the recruiting HDACs. Because Sp1 accumulates in several types of cancer including lung cancer [33], understanding the Sp1 transcriptional regulatory network may provide novel insights into the molecular origins and treatment of lung cancer. In our previous studies of lung cancer, we found that Sp1 was highly upregulated in the early stages of cancer progression, but partially down regulated in the late stages. Our previous studies also showed that regulation of Sp1 protein stability by phosphorylation and sumoylation contributed to its expression in the early and late stages of cancer, respectively [32]. Kras activation and the Notch pathway might activate ERK1/2 to phosphorylate Sp1, thus stabilizing Sp1 in the early stages of cancer [32, 34]. In the late stages, Sp1 could be sumoylated, leading to recruitment of its E3-ligase, RNF4, followed by polyubiquitination and degradation [32]. To clarify the molecular mechanism underlying gene regulation by Sp1, we used microarray analysis to assess gene expression in Kras^G12D^-induced lung tumor transgenic mice and identified thousands of genes potentially regulated by Sp1 [23]. However, some of the genes do not harbor a conserved Sp1 binding motif within their promoter region, suggesting that another regulatory mechanism is involved in Sp1-mediated gene regulation. In this study, we identified a novel pathway for Sp1-mediated activation wherein miR-182 expression downregulated the expression of FOXO3, a known miR-182 target gene [35]. Sp1 activated miR-182 and FOXO3 at the transcriptional level; however, FOXO3 protein expression decreased. These results suggest that post-transcriptional regulation by miRNAs is a powerful mechanism by which to control the final level of protein expression. Many coding genes with Sp1 binding element(s) in their promoters harbor conserved miRNA target sequences in their 3'-UTR. To our knowledge, this is first study to demonstrate that Sp1 regulates the expression of a target gene by regulating promoter activity and post-transcriptional processing in parallel.

Few studies have characterized the regulation of miRNA by Sp1. Herein, using a bioinformatics approach, we identified several miRNAs potentially regulated by Sp1, including miR-182. We then showed that Sp1 specifically targets the miR-182 promoter region and activates miR-182 expression. miR-182 reportedly forms a gene cluster with two adjacent miRNAs (miR-96 and miR-183) [35]. The expression of miR-96 and miR-183 also decreased following Sp1 knockdown (Supplementary Figure S2A). Moreover, we also investigated the binding of Sp1 to the miR-212 promoter because the latter contains 13 putative Sp1 binding sites (Supplementary Table S1). We found that Sp1 bound to the miR-212 promoter sequence (Supplementary Figure S2B and S2C). Interestingly, a recent study showed that FOXO3 is a direct target of miR-212 in the neurons of patients with Alzheimer's disease [36]. miR-182 and miR-212 might cooperate to downregulate FOXO3 expression upon Sp1 overexpression. We cannot rule out this possibility. However, depletion of miR-182 was sufficient to impair the Sp1-mediated reduction of FOXO3 expression in our experiments (Figure 4C), suggesting that miR-182 is the major regulator of FOXO3 in lung cancer cells.

Several studies have shown that miR-182 is upregulated in lung cancer. This suggests that miR-182 plays a positive role in lung tumorigenesis. However, in two studies of miR-182 function in lung cancer, miR-182 inhibited the proliferation of human lung adenocarcinoma cells [37, 38]. Our results in this study provide several pieces of evidence to support the notion that miRNA-182 is a positive regulator of lung cancer cell proliferation. Firstly, miR-182 was upregulated in the majority of lung cancer clinical samples and lung cancer cell lines examined. Secondly, miR-182 knockdown inhibited cell cycle progression and cell growth. Finally, miR-182 knockdown reduced lung tumor growth *in vivo*. Discrepancies in the role of miRNA-182 in lung cancer cell proliferation might derive from the different experimental designs of the studies. For example, because miR-182 expression is upregulated in lung cancer, we knocked down its expression and examined the effects on cancer cell proliferation. However, other studies that described a negative role of miR-182 in lung cancer used miR-182 overexpression to study miR-182's role in cancer cell proliferation. Overexpression conditions can alter the function of many genes [39]. For example, Sp1 accumulates in most of cancers; knockdown of Sp1 expression decreases cell proliferation, but Sp1 overexpression also attenuates cancer cell growth [40]. Because post-translational modifications affect protein function, overexpressed proteins might not be completely processed, which could affect their function. Previous studies in melanoma and hepatocellular carcinoma indicated that miR-182 enhanced tumor metastasis [35, 41]. However, our data, as shown in Figure 6, indicated that miR-182 knockdown altered cell morphology and increased migration and invasion activities. In addition, miR-182 knockdown increased N-cadherin levels, suggesting that miR-182 promotes the mesenchymal to epithelial transition (MET) [42]. Previous studies have shown that TIMP-2 enhances the E-cadherin/β-catenin complex in A549 lung cancer cells [43]. Whether Sp1 or miR-182 regulates TIMP-1 in lung cancer needs to be addressed in future studies. Finally, miR-182 levels were lower in CL1-5 cells then in CL1-0 cells, resulting in increased metastatic activity in CL1-5. Collectively, our data suggest that miR-182 inhibits lung cancer metastasis. Our previous study indicated that Sp1 is down regulated in the late stages of lung cancer progression [32]. Therefore, in the late stages of lung tumorigenesis, miR-182 expression was down regulated, compared with expression in the early stages, which led to tumor metastasis through, at least in part, an increase in FOXO3 expression. It is still not clear why miR-182 has different roles in different types of cancer; this awaits further study.

Although we found that FOXO3 is involved in miR-182-mediated lung cancer progression, FOXO3 knockdown did not completely abolish the effects of miR-182 knockdown, suggesting that other genes regulated by miR-182 contribute to the inhibition of metastasis by miR-182. With this in mind, we determined the expression profile of miR-182-regulated genes. Many metastasis-related genes were induced in miR-182-knockdown cells, including CD44, ADAM9, and CDH9. CD44, which localizes to the cell membrane, is reportedly involved in cell migration in various cancer types [44]. Recent studies also showed that tumor initiating cells with high CD44 expression maintained lung cancer tumorigenicity and drug resistance [45]. Another metastasis-related gene induced by miR-182 knockdown, ADAM9, cleaves membrane proteins such as E-cadherin [46]. A previous study showed that combined Kras and Wnt pathway activation increased the incidence of lung cancer formation [47]. Given that ADAM9 is also involved in the activation of the Wnt pathway, Sp1 and miR-182 might connect the Kras and the Wnt pathway. In addition, CDH9 also involves in the cancer metastasis [48].

In conclusion, we showed that miR-182 is an Sp1-activated miRNA, whose expression increased in lung cancer. miR-182 functioned not only as an oncomiR for lung cancer growth, but also as a suppressor of lung cancer metastasis.

## MATERIALS AND METHODS

### Cell culture and transfection

Human lung cancer cell lines, A549, H1299, CL 1-0 and 1-5, were cultured in Dulbecco's modified Eagle's medium (Invitrogen, Carlsbad, CA), human diploid fibroblasts, IMR, were cultured in Minimum Essential Media (Invitrogen) and human bronchial epithelial cells, BEAS-2B, was cultured in RPMI 1640 Medium (Thermo Scientific, Rockford, IL). All of culture mediums contained 10% fetal bovine serum, 100 U/ml penicillin G sodium and 100 μg/ml streptomycin sulfate (Invitrogen). Cells were cultured at 37℃ and 5% CO_2_. Transfection of all cells with expression vectors was done using Lipofectamine 2000 (Invitrogen) according to the manufacturer's directions.

### Reverse transcription-polymerase chain reaction (RT-PCR) and stem-loop RT-PCR

Total RNA was isolated using the Trizol reagent (Invitrogen), and 3 μg of RNA were reverse-transcribed using the Superscript III enzyme (Invitrogen). PCR was then performed on cDNA with gene-specific primers: Sp1, F, 5'-TGC AGC AGA ATT GAG TCA CC-3' and R, 5'-CAC AAC ATA CTG CCC ACC AG-3'; FOXO3, F, 5'-GCA AGC ACA GAG TTG GAT GA-3' and R, 5'-CAG GTC GTC CAT GAG GTT TT-3'; GAPDH, F, 5'-GAG TCA ACG GAT TTG GTC GT-3' and R, 5'-TTG ATT TTG GAG GGA TCT CG-3'; and U6, F, 5'-CGC TTC GGC AGC ACA TAT AC-3' and R, 5'-AGG GGC CAT GCT AAT CTT CT-3'. The protocol for the detection of mature miRNAs using a stem-loop gene-specific reverse transcription primer was performed as described previously [49]. Stem-loop primers (miR-182, 5'-GTC GTA TCC AGT GCA GGG TCC GAG GTA TTC GCA CTG GAT ACG ACA GTG TG-3'; miR-96, 5'-GTC GTA TCC AGT GCA GGG TCC GAG GTA TTC GCA CTG GAT ACG ACA GCA AA-3'; and miR-183, 5'-GTC GTA TCC AGT GCA GGG TCC GAG GTA TTC GCA CTG GAT ACG ACA GTG AA-3') were designed to specifically reverse transcribe the mature miRNA of interest. The primers for PCR were as follows: miR-182, F, 5'-CGG CGG TTT GGC AAT GGT AGA ACT-3'; miR-96, F, 5'-CGG CGG TTT GGC ACT AGC ACA TTT-3'; miR-183, F, 5'-CGG CGG TAT GGC ACT GGT AGA ATT-3'; and R, 5'-CCA GTG CAG GGT CCG AGG TAT-3'. PCR products were analyzed by ethidium bromide-containing agarose gel electrophoresis.

### Western blotting

Cell lysates were prepared from the indicated cell lines for SDS-polyacrylamide gel electrophoresis (SDS-PAGE), which was then transferred to a polyvinylidene difluoride membrane (Millipore, Billerica, MA) by using a transfer apparatus according to the manufacturer's protocols. Membranes were blocked with 3% nonfat milk in TBST buffer (10 mM Tris-HCl, pH 8.0, 150 mM NaCl and 0.05% Tween 20) for 1 h, washed in the same buffer and incubated with antibodies against Sp1 (Millipore), GFP (Clontech, Palo Alto, CA), FOXO3 (Genetex, Hsinchu, Taiwan), tubulin (Sigma-Aldrich, St. Louis, MO), N-cadherin (Cell Signaling Technology, Beverly, MA), β-catenin (Cell Signaling Technology) and vimentin (Epitomics, Burlingame, CA) at 4℃ overnight. Membranes were washed three times for 10 min and incubated with the secondary antibody (goat anti-rabbit or anti-mouse immunoglobulin G linked with horse radish peroxidase (Millipore)) for 1 h at room temperature. After three more washes, the protein bands were detected with the ECL Western blotting Detection System (Millipore) and recorded with the FluorChem image analysis system (Alpha Innotech, San Leandro, CA). Band intensities were quantified with Scion image software (Scion, Frederick, MD).

### Luciferase reporter assay

Cells were transiently cotransfected with reporter plasmids (pGL2-miR-182, pGL2-miR-182 mutants, pGL3-FOXO3-3'UTR or pGL2-FOXO3) and expression plasmids of interest using Lipofectamine 2000. Luciferase activity in cell lysates was determined by luminometer (LB9506; Berthold Technologies, Bad Wildbad, Germany) and normalized to total protein concentration. For construction, genomic DNA of A431 cells were prepared. The miR-182 promoter (-1000/+50) was produced by PCR using primers: F, 5'-GGG CAG GCA GCC TGC ACC CT-3' and R, 5'-CAC CAG TGT GAG TTC TAC CAT TGC-3'. The FOXO3 (-1000/+50) promoter was produced by PCR using primers: F, 5'-ACG CGT CGA GCT GAC AGG CGG TTC-3' and R, 5'-AGA TCT CGC CCC CCG GCC AGG CCG-3'. After amplification and purification, the DNA fragments were ligated to pGL2 vector using restriction enzymes, KpnI and BglII for pGL2-miR-182, MluI and BglII for pGL2-FOXO3 (New England Biolabs, Ipswich, MA). For construction of pGL3-FOXO3-3'UTR, cDNA of H1299 cells was prepared. The FOXO3 3'UTR was produced by PCR using primers: F, 5'-TCT AGA AGG ATC ACT GAG GAA GGG-3' and R, 5'-TCT AGA TCT GCA AAG CAA AAC AGG-3'. After amplification and purification, the DNA fragments were ligated to pGL3 vector using restriction enzymes, XbaI. For construction of pGL2-miR-182 mutants, the pGL2-miR-182 plasmid was used as the template for mutagenesis of Sp1-binding sites. Primers for mutations of site 1 (-433C/-434G to−433A/-434A): F, 5'-CTT AGT AAA TAG CAA AAC CCA AAC CAC ATT AGC CAT CTC TTC CC-3' and R, 5'- GGG AAG AGA TGG CTA ATG TGG TTT GGG TTT TGC TAT TTA CTA AG-3'; and for site 2 (-398C/-399G to−398A/-399A): F, 5''-CCA GCG CCC AGG GAA AGG GCT CTC TGG C-3' and R, 5'- GCC AGA GAG CCC TTT CCC TGG GCG CTG G−3'. Mutagenesis was performed by PCR using plaque-forming unit DNA polymerase (Agilent Technologies, Santa Clara, CA).

### Chromatin immunoprecipitation (ChIP) assay

The protocol for ChIP was performed as described previously [23]. Briefly, formaldehyde-fixed DNA–protein complex was immunoprecipitated with 5 mg of normal rabbit IgG, anti-acetyl-Histone H3 (Millipore), or anti-Sp1 antibodies. Immunoprecipitated DNA was analyzed by PCR. The primer sequences for promoter of miR-182 in PCR analyses were as follows: F, 5'-ACT TCC CTC TCT CCC TTT GG-3' and R, 5'-CAC CTG ACA GCA GGG ACT CA-3'. The primer sequences for promoter of miR-212 in PCR analyses were as follows: F, 5'-AGC GGA GCT GTC CTC TCA G-3' and R, 5'-CCG GGC AGT AAG CAG TCT A-3'. The primer sequences for promoter of p21 in PCR analyses were as follows: F, 5'- ACC AAC GCA GGC GAG GGA CT-3' and R, 5'- CCG GCT CCA CAA GGA ACT GA−3'.

### DNA affinity precipitation assay (DAPA)

The DNA oligonucleotides (miR-182, 5'-AAA ACC CAG CCC ACA TTA GCC ATC TCT TCC CCA GCG CCC AGG GGC AGG GCT CT-3'; miR-212, 5'-GAC CGG GGG GGC GGG GCC TCC CAG GTC CCG CCC CGC CCC CAC GCC CCC GCC GG-3'; and p21, 5'- CCC GCC TCC TTG AGG CGG GCC CGG GCG GGG CGG-3') were biotinylated at 5' end and then annealed with their complementary strands. The assay was performed by incubating 1 μg of biotin-labeled probe with cell extract in 1 ml of DAPA buffer (60 mM KCl, 12 mM HEPES, pH 7.9, 4 mM Tris-HCl, 5% glycerol, 0.5 mM EDTA and 1 mM dithiothreitol). After incubation for 1 h at 4℃, DNA–protein complexes were then incubated with 20 μl of streptavidin-agarose (Sigma-Aldrich) for 1 h at 4℃. DNA–protein complexes were then washed three times in the DAPA buffer.

### Clinical specimens of patients with lung adenocarcinoma

Human clinical specimens used in this study were approved by the Clinical Research Ethics Committee at the Medical Center of National Cheng Kung University (Tainan, Taiwan). After surgical resection at National Cheng Kung University Hospital, specimens of patients with lung adenocarcinomas were collected for immunohistochemical analysis, RT-PCR or Western blotting.

### shRNA lentivirus production

We purchased scramble, Sp1 and FOXO3 shRNA from National RNAi Core Facility in Academia Sinica of Taiwan (Taipei, Taiwan) and miRZip and miRZip-182 from SBI (System Biosciences, CA). The lentiviruses were obtained from RNAi Core of Research Center of Clinical Medicine, National Cheng Kung University Hospital. (The protocol is described below-293T cells were cotransfected with 5 μg of packaging plasmid (pCMVΔR8.91), 0.5 μg of envelop plasmid (pMD.G) and 5 μg of pLKO.1 shRNA using Lipofectamine 2000 for 6 h. After 24 h incubation, the supernatants containing viral particles were harvested and filtered through 0.45 mm filters.)

### Fluorescence-activated cell sorting (FACS)

The miRZip and miRZip-182 stable expression H1299 cells were washed with PBS and fixed in cold 70% ethanol overnight at 4 Cells were then washed with cold PBS and permeabilized with 0.1% Triton X-100 for 10 min. After treatment with 10 μg/ml RNase A (Qiagen, Germantown, MD) at 37 for 1 h, cells were stained with 50 μg/ml propidium iodide (Sigma-Aldrich) at room temperature for 2 h. Finally, cells were analyzed by flow cytometer on the FACSCalibur (BD Biosciences, Franklin Lakes, NJ).

### Xenograft study

The animal experiment was approved by the Institutional Animal Care and Use Committee at National Cheng Kung University. Female SCID mice were purchased from National Laboratory Animal Center in Taiwan. The miRZip and miRZip-182 stable expression H1299 cells (10^6^ cells) were suspended in 100 μl of PBS and implanted into the back of SCID mice.

### Immunohistochemistry (IHC)

The experimental process of IHC was performed as described in our previous study [32]. Briefly, blocked histological sections were stained with the anti-Sp1 or anti-FOXO3 antibodies. The immunoreactivity was detected by a Vectastain ABC kit (Vector Laboratories, Burlingame, CA).

### Quantitative PCR

Quantitative Real-time PCR was performed using SYBR Premix Ex Taq (Takara Bio, Otsu, Shiga, Japan) in a CFX96TM Real-Time System and C1000 TM Thermal Cycler (BIO-RAD, Hercules, CA). The primers for quantitative PCR were as follows: Firefly luciferase, F, 5'-TCA AAG AGG CGA ACT GTG TG-3', R, 5'-GGT GTT GGA GCA AGA TGG AT-3'.

### Immunofluorescent staining

The miRZip and miRZip-182 stable expression H1299 cells were seeded onto coverslips (with a thickness of 0.17 mm) and incubated for 24 h. After fixation with 4% paraformaldehyde (Sigma-Aldrich) in PBS for 15 min and permeabilization with 1% Triton X-100 for 5 min, cells on the coverslip were blocked with 1% bovine serum albumin (Sigma-Aldrich) for 1 h and stained with the antibody against F-Actin by using Alexa Fluor 568-conjugated phalloidin (Invitrogen) for 1 h at room temperature. Subsequently, cells on the coverslip were washed with PBS three times. Finally, cells were mounted in Prolong Gold antifade reagent with DAPI (Invitrogen), and examined using immunofluorescence microscope (Delta Vision Personal DV). The images were analyzed with softWoRx software (Applied Precision).

### Wound-healing assay

The miRZip and miRZip-182 stable expression H1299 cells (1.5 x 10^6^) were seeded in 6 cm dish and cultured for 24 h, the linear wound of cellular monolayer was created by scratching confluent cell monolayer using a plastic pipette tip. The monolayer of Scratched cell was washed by PBS to remove debris. After incubation at 37℃ with 5% CO_2_ for 16 h, area of migration was photographed under light microscope for evaluation.

### Transwell migration assay

The cell migration assay was performed using Transwell system with an 8 mm pore size polycarbonate filter membrane (Corning Costar, Cambridge, MA). Cells were trypsinized and suspended in serum-free DMEM. Upper wells were filled with cell suspensions in serum-free DMEM and lower wells were filled with DMEM containing 10% fetal bovine serum. After incubation for 14 h at 37℃ with 5% CO_2_, the lower side of filter membrane was fixed with methanol and stained with DAPI. The migrated cells were counted under a fluorescent microscope and quantified by Image J software (National Institutes of Health, Bethesda, MD).

### In vivo metastasis assay

The miRZip and miRZip-182 stable expression H1299 cells were trypsinized and suspended in PBS for tail vein injection. A total of 4 x 10^6^ cells in 100 ml of PBS were injected into the lateral tail vein of SCID mice. Mice were killed after 8 weeks, and the excised lungs were fixed with 4% formaldehyde for 48 h. Finally, the number of pulmonary metastatic nodules on the surface of lung was counted.

### Microarray analysis

Total RNA was extracted using TRIzol from miRZip and miRZip-182 stable expression H1299 cells. Microarray analysis was performed by the Phalanx Biotech Group (Hsinchu, Taiwan). Microarray data were analyzed by using DAVID Bioinformatics Resources 6.7 [50, 51].

## SUPPLEMENTARY TABLES, REFERENCES AND FIGURES


