# Supplementary material for: Sp1-mediated microRNA-182 expression regulates lung cancer progression
Source: Oncotarget. 2014 Jan 25;5(3):740–53. doi: 10.18632/oncotarget.1608 (PMC3996653; doi:10.18632/oncotarget.1608)
Supplement: Supplementary file 1 [file oncotarget-05-740-s001.pdf]

Supplementary data

Sp1-mediated microRNA-182 expression regulates lung cancer progression

Wen-Bin Yang, Ping-Hsin Chen, Tsung-I Hsu, Tzu-Fun Fu, Wu-Chou Su, Hungjiun Liaw, Wen-Chang Chang and Jan-Jong Hung

Supplementary Table S1

Supplementary References (52-58)

Supplementary Figure Legends S1 – S3

Supplementary Tables S1. miRNAs differentially expressed between lung cancer and normal lung tissues, which were potentially regulated by Sp1

miRNA	Number of putative Sp1 binding sites	Location	Ref.
Up regulated in lung cancer			
hsa-mir-20b	1	Xq26.2	[52]
hsa-mir-24-2	2	19p13.13	[53]
hsa-mir-106a	1	Xq26.2	[52, 53]
hsa-mir-150	3	19q13.33	[53]
hsa-mir-182	2	7q32.2	[52, 54-57]
hsa-mir-183	2	7q32.2	[52, 55, 56]
hsa-mir-192	3	11q13.1	[53]
hsa-mir-193a	5	17q11.2	[57]
hsa-mir-200a	3	1p36.33	[52, 57]
hsa-mir-200b	9	1p36.33	[54, 58]
hsa-mir-203a	9	14q32.33	[52-54]
hsa-mir-212	13	17p13.3	[53]
Down regulated in lung cancer			
hsa-mir-29b-2	1	1q32.2	[53]
hsa-mir-30b	1	8q24.22	[58]
hsa-mir-138-1	1	3p21.32	[54, 58]
hsa-mir-143	1	5q32	[53]
hsa-mir-145	1	5q32	[53-55]
hsa-mir-181c	3	19p13.13	[53]
hsa-mir-516a-1	2	19q13.42	[58]
hsa-mir-516a-2	1	19q13.42	[58]
hsa-mir-520h	1	19q13.42	[58]
hsa-mir-521-1	1	19q13.42	[58]

Supplementary References

52. Raponi M, Dossey L, Jatkoe T, Wu X, Chen G, Fan H and Beer DG. MicroRNA classifiers for predicting prognosis of squamous cell lung cancer. Cancer Res. 2009; 69(14):5776-5783.
53. Yanaihara N, Caplen N, Bowman E, Seike M, Kumamoto K, Yi M, Stephens RM, Okamoto A, Yokota J, Tanaka T, Calin GA, Liu CG, Croce CM and Harris CC. Unique microRNA molecular profiles in lung cancer diagnosis and prognosis. Cancer Cell. 2006; 9(3):189-198.
54. Tan X, Qin W, Zhang L, Hang J, Li B, Zhang C, Wan J, Zhou F, Shao K, Sun Y, Wu J, Zhang X, Qiu B, Li N, Shi S, Feng X, et al. A 5-microRNA signature for lung squamous cell carcinoma diagnosis and hsa-miR-31 for prognosis. Clin Cancer Res. 2011; 17(21):6802-6811.
55. Cho WC, Chow AS and Au JS. Restoration of tumour suppressor hsa-miR-145 inhibits cancer cell growth in lung adenocarcinoma patients with epidermal growth factor receptor mutation. Eur J Cancer. 2009; 45(12):2197-2206.
56. Jang JS, Jeon HS, Sun Z, Aubry MC, Tang H, Park CH, Rakhshan F, Schultz DA, Kolbert CP, Lupu R, Park JY, Harris CC, Yang P and Jen J. Increased miR-708 expression in NSCLC and its association with poor survival in lung adenocarcinoma from never smokers. Clin Cancer Res. 2012; 18(13):3658-3667.
57. Ma L, Huang Y, Zhu W, Zhou S, Zhou J, Zeng F, Liu X, Zhang Y and Yu J. An integrated analysis of miRNA and mRNA expressions in non-small cell lung cancers. PLoS One. 2011; 6(10):e26502.
58. Seike M, Goto A, Okano T, Bowman ED, Schetter AJ, Horikawa I, Mathe EA, Jen J, Yang P, Sugimura H, Gemma A, Kudoh S, Croce CM and Harris CC. MiR-21 is an EGFR-regulated anti-apoptotic factor in lung cancer in never-smokers. Proc Natl Acad Sci U S A. 2009; 106(29):12085-12090.

Supplementary Figures

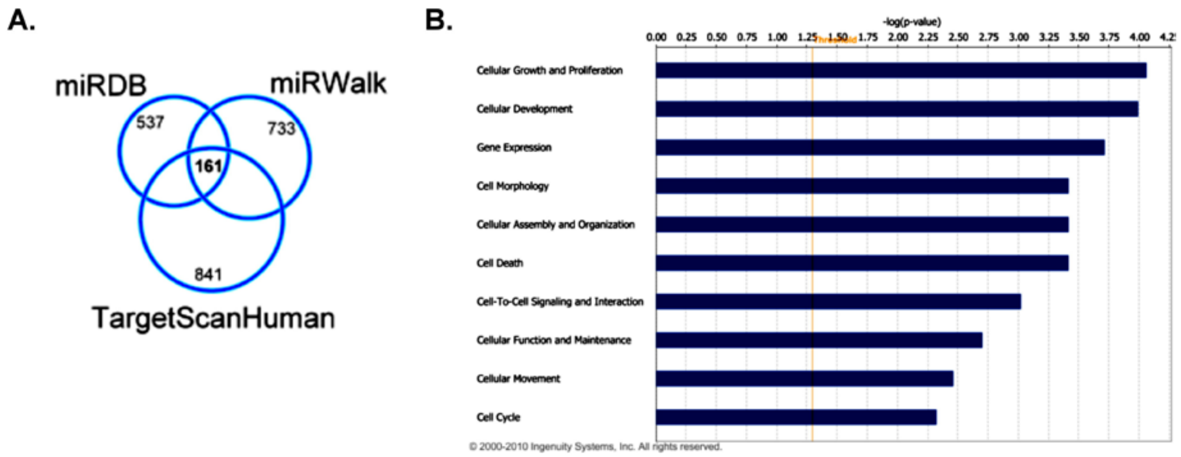

Supplementary Figure S1. Bioinformatics analysis reveals potential target genes of miR-182. (A) The miRNA target prediction databases (miRDB, miRWalk, TargetScanHuman) were used for theoretical target genes prediction. There are 161 genes highly potential regulated by miR-182. (B) Pathway analysis by Ingenuity software was conducted on 161 potential target genes to evaluate the miR-182 function.

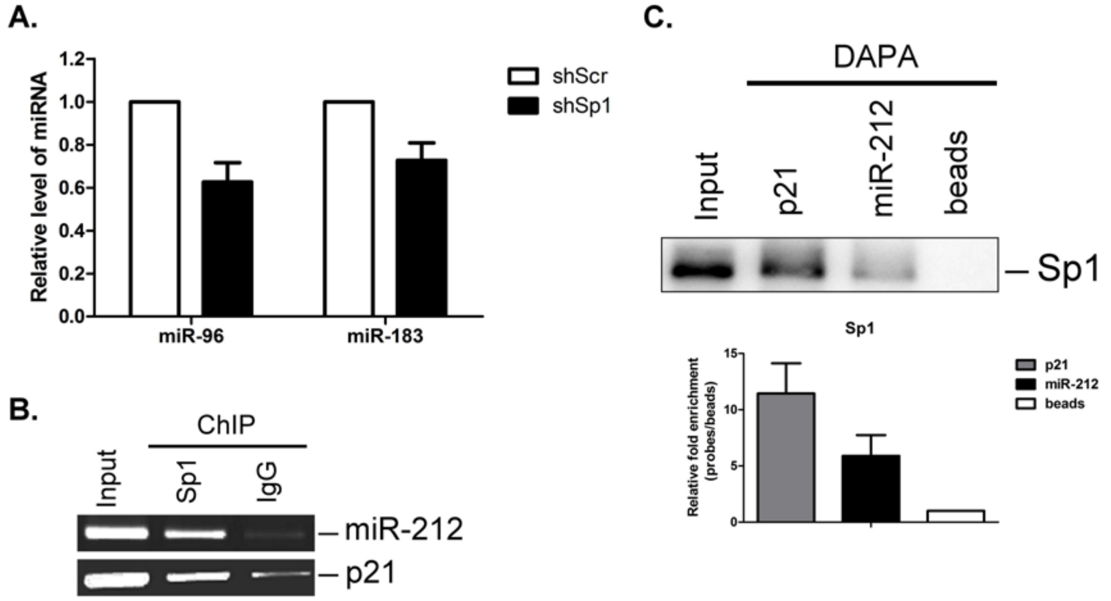

Supplementary Figure S2. miR-96, miR-183 and miR-212 are regulated by Sp1. (A) Scramble and Sp1 shRNAs were transfected into H1299 for 48 h, and miR-96 and miR-183 mRNA were determined by quantitative RT-PCR. (B) A549 cells were fixed with 1% formaldehyde, and ChIP assays were performed using anti-Sp1 antibodies. DNA was then

extracted from the sample for PCR using primers for the miR-212 and p21 promoter. (C) A549 cells were harvested, lysed, and prepared for DAPA with a biotin-conjugated p21 and miR-212 promoter probes. Samples were analyzed by Western blot using anti-Sp1 antibody. Data are representative of three independent experiments. Quantitative results are shown in the bottom lower panel.

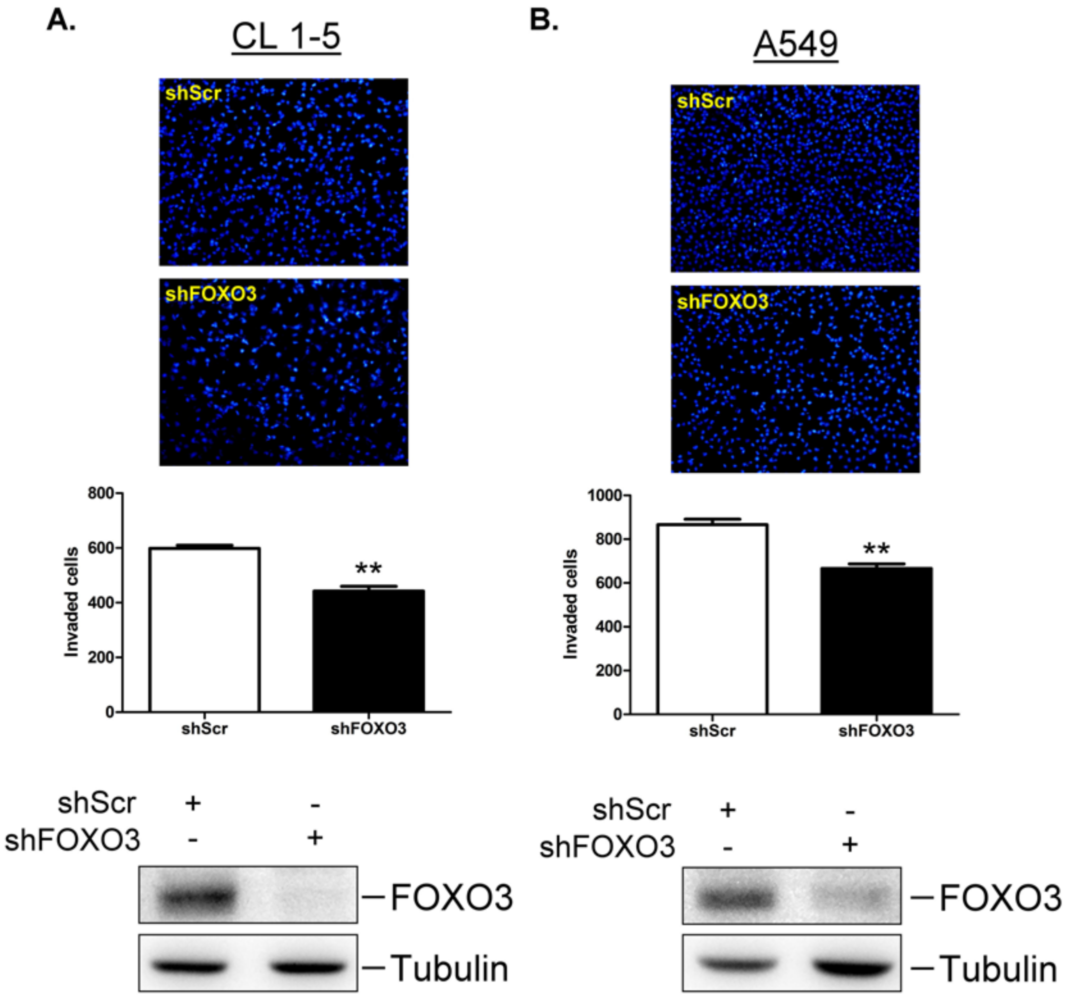

Supplementary Figure S3. Migration assay was performed with Transwell chanbers in FOXO3 knockdown CL1-5 (A) and A549 (B). Data are representative of three independent experiments and presented as the mean ± SEM. The level of statistical significance was determined by t-test (**, $p<0.01$).
